# Evidence of physiological changes associated with single-session pre-frontal tDCS: a pilot study

**DOI:** 10.3389/fnhum.2025.1549248

**Published:** 2025-02-25

**Authors:** Hannah N. Rembrandt, Ellyn A. Riley

**Affiliations:** Aphasia Lab, Department of Communication Sciences and Disorders, Syracuse University, Syracuse, NY, United States

**Keywords:** aphasia, tDCS, pupillometry, EEG, attention

## Abstract

**Objective:**

Transcranial direct current stimulation (tDCS), a non-invasive, painless method of applying direct current electrical stimulation to specific areas of the brain, is an effective method for enhancing attention and post-stroke fatigue, as shown by behavioral improvements in post-stroke populations. While behavioral evidence supports this method, there is a paucity of physiological data corroboration of this improvement. The current study is designed to investigate if a single session of tDCS will improve attention and fatigue as shown by relevant physiological methods in persons with post-stroke aphasia.

**Methods:**

Ten participants (5 male; mean age: 62.8) engaged in two identically structured data collection sessions with at least a 3-day wash-out period between them. Sessions started with a sustained attention task with simultaneous electroencephalography (EEG) and pupillometry data collection, followed by an attention training program with simultaneous active or sham tDCS. Following tDCS, participants repeated the sustained attention task with simultaneous EEG and pupillometry data collection. Participants received active tDCS during one session, and sham tDCS during the other, with the order randomized.

**Results:**

No differences between conditions were found for either behavioral results from the sustained attention task (i.e., reaction time of correct responses; *n* = 9 *p* = 0.39) or EEG measured attention state data for any of the four attention states: no attention (*n* = 10, *p* = 0.83), distracted attention (*n* = 10, *p* = 0.20), moderate attention (*n* = 10, *p* = 0.95), or high attention (*n* = 10, *p* = 0.62). Pupil dilation was significantly greater in the post-active tDCS stimulation condition than in either pre-training condition (*n* = 10, *p* < 0.01). tDCS stimulation lessened the increase in task-based fatigue from the beginning to the end of the session such that there was a significant increase in task-based fatigue when participants received sham tDCS (*n* = 10, *p* = 0.01) but no significant change in task-based fatigue during the active condition session (*n* = 10, *p* = 0.12).

**Conclusion:**

Changes in pupil diameter observed in the active stimulation condition suggest activation of the locus coeruleus-norepinephrine (LC-NE) pathway within a single session of tDCS administration, but the lack of significant changes for either response time or attention states indicate no direct effect on behaviorally measured or EEG measured attention within the same timeframe. Responses to active stimulation in terms of subjective fatigue rating varied between individual participants; overall, active tDCS mitigated task-based fatigue. More research is needed to investigate this relationship.

## 1 Introduction

Aphasia, an acquired language disorder affecting language expression and comprehension, occurs secondary to an injury to the brain such as stroke, typically in the left hemisphere (American Speech-Language-Hearing Association, n.d.). Language is the primary source of deficit that affects persons with aphasia (PWA). However, there is evidence suggesting that co-occurring cognitive deficits, such as attention, are common within this population (Murray, 2012). While incidence of fatigue in PWA is unclear, potentially due to a noted lack of PWA representation in research about fatigue (Riley et al., 2021), pooled prevalence of post-stroke fatigue independent of aphasia diagnosis is approximately 50% (Cumming et al., 2016; Zhan et al., 2022).

The concomitance of cognitive deficits that PWA experience affects their language skills and recovery through the interrelatedness of attention and language. Studies have shown that impairments in attention may be associated with impairments in language, and that therapy including attentional skills in its treatment targets can improve language outcomes (Varkanitsa et al., 2023).

Additionally, fatigue has been shown to impact cognitive skills (Almhdawi et al., 2021; Pihlaja et al., 2014; Shetty et al., 2023) and may have a mutually impactful relationship with speech and language disorders (Riley et al., 2021). This illuminates the need for effective treatments addressing attention and fatigue which can be used in conjunction with language and other cognitive therapies with the aphasia population.

One adjuvant therapeutic tool growing in prevalence in research is transcranial direct current stimulation (tDCS), a non-invasive method of neuromodulation via electrical stimulation applied to a targeted region of the brain. Evidence supports the use of tDCS in language therapy with PWA when targeting regions of the brain associated with motor speech and language, specifically regions in the frontotemporal area (Jung et al., 2011; Monti et al., 2013). Similarly, behavioral evidence has shown improvements in attention when tDCS is applied to brain regions associated with cognitive skills (Kang et al., 2009; Kazinka et al., 2024; Martin et al., 2023), including the dorsolateral prefrontal cortex (DLPFC) or the posterior parietal cortex. Applying tDCS to the motor cortex has also shown improvements in fatigue (De Doncker et al., 2021).

The current study is investigating the impact of tDCS on the DLPFC on attention, as this brain region has been found to be relevant specifically to attention bias (Knight et al., 2020), including visual emotional attentional bias (Wang et al., 2024), and top-down cognitive control of task-related attentional processes (Brosnan and Wiegand, 2017).

Research on the lateralization of the DLPFC has revealed disparate findings (Brosnan and Wiegand, 2017); however, tDCS studies investigating the differences between the left and right DLPFC in attentional control have suggested that the left DLPFC is more associated with selective visual attention (Hertrich et al., 2021; Spooner et al., 2020).

Both anodal and cathodal tDCS has been shown to reduce distraction during an attention task when targeting the left DLPFC, with anodal tDCS associated specifically with increased cognitive control when ignoring irrelevant stimuli (Knight et al., 2020).

With regard to emotional attentional bias, the left DLPFC has been shown to be associated with a reduction in both attentional bias and reactivity to emotional stimuli (Wang et al., 2024) with reactivity to negative emotional stimuli further attenuated by active tDCS as compared to sham (Clarke et al., 2020). Conversely, pupil dilation has been impacted by the emotional valence of presented stimuli during tDCS administration on the DLPFC, with stimulation to the left DLPFC shown to increase pupil dilation only when presented with negative emotional stimuli (Allaert et al., 2019).

While direct impacts of tDCS on language are outside the scope of the current study, the DLPFC has also been shown to interact with other areas of the brain when an individual is engaging in discourse-level language processing and production (Hertrich et al., 2021) and cathodal tDCS applied to the left DLPFC showed changes in reaction time during both language comprehension (i.e., an increase in reaction time) and production (i.e., a decrease in reaction time) providing support for the left DLPFC’s involvement in language (Klaus and Schutter, 2018). This has also been shown in PWA, with evidence supporting the role of the left DLPFC in grammar comprehension, demonstrated via improvements in incorrect grammar judgement following active tDCS targeting this region (Riley et al., 2022).

While there are many studies demonstrating behavioral evidence supporting tDCS as an effective method for treating attention and other cognitive skills, there are considerably fewer providing the relevant physiological evidence (Dubreuil-Vall et al., 2019). Attention is commonly measured physiologically via electroencephalography (EEG; Liu et al., 2013; Riley and Owora, 2020) and pupillometry (Strauch et al., 2022; Zhao et al., 2019).

EEG measures electrical activity fluctuations on the surface of the scalp, serving as an effective physiological measure for cognitive behaviors such as attention (Myrden and Chau, 2016). With strong temporal resolution, EEG is also well suited to monitor real-time fluctuations in attention during a cognitive task (Kaushik et al., 2022).

Non-light mediated pupil dilation has long been connected with cognition (Beatty, 1982). The use of pupillometry as a physiological measurement for cognitive skills such as attention has grown in research due to its use as a proxy measurement for locus coeruleus activation (Reilly et al., 2024c). The locus coeruleus is the main hub for norepinephrine production in the brain (Poe et al., 2020) and the locus coeruleus-norepinephrine (LC-NE) pathway has been long associated with arousal (Huang et al., 2023; Lenartowicz et al., 2013) and attention (Bouret and Sara, 2004; Harley, 1991; McBurney-Lin et al., 2019). Norepinephrine also regulates pupil size such that activation of the LC dilates the pupil and inhibition of the LC leads to pupil constriction (Viglione et al., 2023) likely due to an inhibitory effect on the parasympathetic oculomotor complex (Laeng et al., 2012).

Post-stroke fatigue can be defined as a potentially overwhelming lack of energy not impacted by rest, which can impact an individual’s physical, emotional, and cognitive well-being (English et al., 2024). Fatigue negatively impacts a person’s quality of life in numerous ways (Almhdawi et al., 2021; Bullier et al., 2020), including by limiting social participation (Quique et al., 2023), a factor self-reported by PWA as a major contributor to their quality of life (O’Halloran et al., 2023).

Fatigue also plays a specific role in attention, with evidence showing that individuals with post-stroke fatigue may have a harder time with sustained attention tasks (Ulrichsen et al., 2020) as well as post-stroke fatigue being associated with difficulty with attention and executive functioning (Pihlaja et al., 2014).

This emphasizes a significant need for efficacious treatment options for fatigue, an area of research that is growing, but still preliminary (English et al., 2024). tDCS is one avenue of fatigue treatments being pursued in research, with preliminary results in multiple populations revealing positive trends in countering fatigue (Ashrafi et al., 2020; De Doncker et al., 2021; McIntire et al., 2017; Lefaucheur et al., 2017b), while also denoting a need for more robust research in this area.

One barrier to research in fatigue treatments is the difficulty in fatigue reporting and assessment (Nadarajah and Goh, 2015), a difficulty compounded for PWA due to the high language demands of commonly used fatigue reporting tools (Riley et al., 2021; Rose et al., 2011). One such example is the Fatigue Severity Scale (FSS; Krupp et al., 1989), a tool with strong reliability and validity within the stroke population (Ozyemisci-Taskiran et al., 2019; Taghizadeh et al., 2024) and recommended to be the primary measure for assessing post-stroke fatigue (English et al., 2024).

To address the accessibility limitations of existing fatigue measures, our research team developed a new fatigue measurement tool that offers an alternative option to the original FSS (Rembrandt et al., 2023). This new measure increases accessibility for PWA by including images with written test items, using simplified language, and allowing for responses in multiple modalities. The first iteration of this tool was used in the current study and has shown strong reliability and validity within healthy and post-stroke populations (Riley et al., 2024b; Riley et al., 2024a). Now known as the Fatigue Interference and Severity Scale for Aphasia (FISS-A; Riley et al., 2025), the final version of this tool further simplifies the language of the test items to be more appropriate for individuals with moderate-to-severe aphasia and provides detailed administration and scoring instructions for clinicians (Riley et al., 2025).

The primary aim of this study was to investigate the effects of a single session of tDCS (paired with a behavioral attention training task) on sustained attention in individuals with post-stroke aphasia, measured using behavioral data (accuracy and reaction time) and physiological data (pupillometry and EEG). The second aim of this study was to examine the impact of a single session of tDCS on subjective ratings of fatigue.

## 2 Methods

### 2.1 Participants

All participants were individuals with post-stroke aphasia at least 6 months post onset. Participants were recruited over the course of six months through the Syracuse University Aphasia Lab’s database of previous research participants. This study was approved by the Institutional Review Board (protocol 18–152) at Syracuse University (SU IRB).

#### 2.1.1 Inclusion and exclusion criteria

Participants were eligible for this study if they met all the following criteria: (1) a diagnosis of aphasia following a stroke, (2) at least 6 months post stroke with no occurrence of seizures during that period, (3) the ability to understand and follow task-specific auditory or written directions.

Participants were ineligible for participation if they met any of the following criteria: (1) a diagnosis of other neurological or psychological conditions (excluding depression or anxiety), (2) past surgery on their head or neck, excluding dental surgery, and/or having metal implants in their head, neck, or chest, (3) presence of a skull fracture, (4) MRI scans revealing a lesion in the targeted tDCS region; i.e., the left dorsolateral prefrontal cortex, as tDCS is ineffective when applied to a lesion.

As these same criteria were applicable to a larger study conducted by this lab, for which all participants were included, none of the recruited participants were excluded from the current study.

#### 2.1.2 Demographics

Ten participants (5 male) with an average age of 62.8 years (range: 39–75) were included in this study; one additional participant was recruited but withdrew prior to the first session.

Participants were all at least 6 months post-stroke, and there were no reported changes in medications or stroke recovery progression between sessions during the present study.

All participants had a diagnosis of post-stroke aphasia, confirmed by language and cognitive testing using the Western Aphasia Battery – Revised (WAB-R; Kertesz, 2007) and the Cognitive Linguistic Quick Test Plus (CLQT+; Helm-Estabrooks, 2001). Testing was conducted as part of a larger study and scores from those sessions were used to determine eligibility for this study. Scores from both of these tests, including the aphasia quotient and subtype from the WAB-R and both the non-linguistic and linguistic cognition ratings, are presented in Table 1.

**Table 1:** Participant demographic information.

Participant	Age	Average FISS-A score	WAB-R aphasia quotient	WAB-R aphasia type	CLQT+ non-linguistic cognition rating	CLQT+ linguistic cognition rating
P8	63	4.56*	97.2	Anomic	WNL	Mild
P9	67	4.67*	94.2	Anomic	WNL	Mild
P10	68	2.78	99.4	Anomic	Mild	WNL
P11	71	1.67	96.8	Anomic	WNL	Mild
P12	62	3.00	50.5	Broca’s	Mild	Moderate
P13	75	2.44	93.4	Anomic	WNL	Mild
P14	61	3.44	40.2	Broca’s	Severe	Moderate
P15	64	3.11	71.1	Conduction	WNL	Mild
P16	39	5.33*	88.8	Anomic	WNL	WNL
P18	58	1.00	27.2	Global	Mild	Severe

WAB-R = Western Aphasia Battery—Revised. CLQT+ = Cognitive Linguistic Quick Test Plus. WNL = Within Normal Limits FISS-A = Fatigue Interference and Severity Scale for Aphasia. * indicates a score above 4.00 denoting clinical fatigue. Mean age: 62.8; mean WAB-R aphasia quotient: 75.88. The Aphasia quotient is a summary score that denotes overall language impairment severity. A lower score indicates more severe impairment.

All participants were administered the FISS-A, to assess their overall post-stroke fatigue. In previous research, participants were considered to experience clinically significant fatigue if they achieved a score of 36 or above (Taghizadeh et al., 2024); converting that sum score to an average across 9 items indicates a threshold of 4.00 as a marker of clinically significant fatigue. Three participants (P8, P9, and P16) showed an average FISS-A score above this threshold.

### 2.2 Materials

#### 2.2.1 Measurement materials

##### 2.2.1.1 Electroencephalogram (EEG)

EEG measurements were collected using a B-Alert^®^ X24t EEG system (Advanced Brain Monitoring). The EEG electrodes were attached to a Velcro^®^ band around the participant’s forehead with plastic straps ensuring proper electrode placement and electrode conduction gel to aid conductivity. At the beginning of the first data collection session, participants underwent specifically designed tasks to calibrate the EEG signal detection to each participant’s unique neural response system. Calibration consisted of three tasks, beginning with a 3-choice psychomotor vigilance task, followed by an eyes open passive vigilance task, and lastly an eyes closed passive vigilance task.

The 3-choice psychomotor vigilance task consisted of three shapes presented at random intervals in random positions of the computer screen. The participants were instructed to respond either “yes” or “no” via right and left arrow key presses, respectively, depending on the shape seen. Passive vigilance was measured through both the second and third calibration tasks by instructing the participants to press the computer keyboard’s space bar every 2 s for 5 min. During the eyes open passive vigilance task, participants were tasked with pressing the space bar each time a red circle flashed on screen, which occurred for 200 ms at 2 s intervals. In the eyes closed passive vigilance task, the red circle was replaced with an audible clang with the participant given the same instructions, with the addition of the instruction to keep their eyes closed for the entire 5 min.

Instructions for calibration tasks were presented on screen and explained verbally by the investigator as many times as needed to ensure task comprehension. Calibration tasks were repeated if needed to achieve sufficient definition of the attention and engagement data classes of No Attention (NA), Distracted Attention (DA), Moderate Attention (MA), and High Attention (HA). Sufficient definition was determined by the B-Alert software and communicated following the completion of the calibration tasks, confirming that the software was able to accurately classify the participant’s EEG responses.

The data classes were defined by the B-Alert system, and presented in the output as a probability the participant was experiencing that level of engagement for each epoch during the attention task. B-Alert classifies these engagement data classes via 1 Hz power spectra densities calculated using a four-class quadratic discriminant function analysis using individual EEG signals from differential sites FzPO and CzPO (Be-Alert Systems, 2024).

The data engagement classes are based on brain waves such that they follow the general pattern wherein high to moderate attention is associated with gamma (25–100 Hz), beta (12.5–25 Hz) and alpha (7.5–12.5 Hz) frequencies; distracted attention is associated with theta (3.5–7.5 Hz) frequencies; and no attention, also referred to as sleep onset, is associated with delta (1.5–3.5 Hz) frequencies (Hibshman and Riley, 2024; Riley et al., 2019). Riley and Owora (2020) contains a more detailed description of the classification process.

##### 2.2.1.2 Pupillometry

An Eyelink^®^ 1,000 Plus eye tracking system was used to measure monocular pupil size during a sustained attention task. Pupil data were collected from the right eye with changes in pupil size measured through pupil diameter. Monocular right pupil measurement was selected based on evidence suggesting congruent dilation patterns across eyes (Winn et al., 2018). Mean pupil diameter per trial was reported, and missing data were excluded from analysis.

Pupil diameter data were converted from arbitrary units to millimeters (mm) using Eyelink’s^®^ recommended scaling factor formula. This formula uses an artificial pupil with a known diameter in mm being measured at the same distance from the Eyelink camera as the participants’ eyes. The arbitrary area of the artificial pupil as reported by the Eyelink Data Viewer software is used in conjunction with the predetermined diameter of the artificial pupil to determine the scaling factor for this experimental setup, and this scaling factor is used in the final conversion from arbitrary units to mm for the participants’ pupils.

##### 2.2.1.3 Behavioral assessments

Behavioral assessment included measures of both attention and task-based fatigue.

A sustained attention task, The Conners Continuous Performance Test-3 (CPT-3; Conners, 2014), was presented at the beginning and end of both sessions. Behavioral data regarding reaction time was collected during this task. The CPT-3 is a visually presented oddball task wherein the participants were instructed to maintain gaze in the center of the computer screen and respond (i.e., press the spacebar on the computer keyboard) when the standard stimuli were shown on screen. The standard stimuli were operationalized as any letter except “X,” and instructions for this task were presented both as text on the screen and as read aloud by the investigator. The instructions were explained as many times and in as much detail as needed to ensure comprehension.

Participants pressed the space bar with their preferred hand. This was their dominant hand for the majority of participants; however, one participant used their non-dominant hand (left) as their right was impacted by hemiplegia secondary to their stroke. This participant expressed no difficulty in pressing the space bar with their non-dominant hand.

The CPT was structured in 6 blocks of 60 trials each, with each trial defined as a single letter presentation. Blocks were split into three subsections defined by the interstimulus intervals (ISI), which included 1,000 ms, 2000 ms, and 4,000 ms. ISI presentation was randomized between blocks.

To measure change in task-based fatigue over the length of the session, participants were asked to verbally rate their current level of fatigue on a scale of 0 to 100, with 0 representing no fatigue, at both the beginning and the end of each session, a method that has demonstrated good validity and reliability (Lee et al., 1991; Tseng et al., 2010). Participants were provided help with conceptualizing this question using a visual scale if needed or further explanation by the researcher to ensure comprehension of the question and their response. Participants’ responses were rated as being appropriate using clinical judgement from a licensed speech-language pathologist.

#### 2.2.2 Training materials

##### 2.2.2.1 Transcranial direct current stimulation

TDCS was administered using a 1×1 transcranial electrical stimulation device (Soterix). Electrode placement was standardized using a Soterix SNAPstrap^™^ and carbon rubber electrodes were covered by a pre-salinated sponge. The anode was placed over the left DLPFC (corresponding to F3 as defined by the international 10–20 EEG system) and the cathode was placed over the contralateral supraorbital region (corresponding to Fp2).

This tDCS montage was selected to target the left DLPFC, and the specific placement of the anode and cathode were selected in accordance with previous literature (Lefaucheur et al., 2017a).

During active stimulation, the current was ramped up to 2 mA in 30 s and maintained at that current threshold for 20 min. During sham stimulation, the current was ramped up to 2 mA followed by an immediate ramp down to 0 mA over the course of a minute. This was done to simulate the sensation of active tDCS administration to blind participants to the stimulation condition.

All participants received both active and sham stimulation across the two sessions, with one condition administered per session. The order of condition presentation was randomized through the use of two identical devices, one programmed to administer active stimulation, and the other programmed to administer sham stimulation. This senior author programmed each device and listed the device order on REDCap so that the data collector would know which device to use in each session. This process ensured that both the data collector and the participant were adequately blinded to the session’s stimulation condition.

##### 2.2.2.2 Attention training

Following each session’s pre-testing CPT task, the EEG electrodes were removed and tDCS electrodes were placed (anode over F3, cathode over Fp2, see section 3.2.3). The training part of the session involved the participants engaging in attention training tasks with simultaneous active or sham tDCS administration. Attention tasks were presented using Attention Process Training – 3 (APT-3; Sohlberg and Mateer, 2010), a computerized attention training program developed to improve attention for children and adults with either developmental or acquired neurological conditions (Sohlberg and Mateer, 2010). The training lasted approximately 25 min across 5 tasks that focused on training sustained and selective attention.

The initial three tasks focused on sustained attention: a single noise identification task, a 2-noise identification task, and a 2-back memory task. Following these three tasks, the single noise identification task and the 2-back memory task were conducted again with auditory distractors to target selective attention. Each task was preceded by verbally and visually presented instructions through the computerized program. Participants were given the option to repeat the instructions as many times as needed to understand the task.

The single and 2-noise identification tasks consisted of directing participants to listen for either 1 or 2 specific sounds in a sound field of categorically related noises (e.g., sounds such as a phone or school bell ringing, or sounds such as an elephant trumpeting or cat meowing). Participants were instructed to either press the spacebar or click a button with the computer mouse when they heard the target sound, which varied by task presentation. The 2-back memory task consisted of showing participants a series of images and directing them to either press the spacebar or click a button with the computer mouse when the image on the screen matched the picture they saw 2 items prior.

Both sessions included the same attention tasks with different stimuli, one session using animal pictures and animal noises, and the other using abstract shapes and everyday noises (e.g., phones ringing, alarms, etc.). The stimuli presentation order (animal or non-animal) across sessions were counterbalanced.

Immediately following each task, the participants were shown a visual representation of their task performance in a graph with correct responses in green and incorrect responses in red, followed by two self-rating questions. The questions asked about effort (i.e., “How hard did your brain work on that exercise?”) and motivation (i.e., “How motivated were you to complete that exercise?”); participants responded by moving a marker along a scale with a range of 0 to 10 wherein 10 represented the most effort and motivation. The scale was supplemented by images of emoji-style faces at different points along the scale to aid in comprehension. These measures of effort and motivation were intended to be part of the training, using metacognition to improve attention in the training tasks (Sohlberg and Mateer, 2010). Specifically, these questions were included as a form of self-monitoring, with the goal of increasing each participant’s self-awareness of their attention difficulties, so as training continued they will be better at effective cognitive resource allocation (Sohlberg and Mateer, 2010). Responses to these questions are not stored or reported, rather they are solely presented for the participant to guide their engagement as they continue through the training program.

### 2.3 Procedure

This study was structured as a within-participant crossover design wherein all participants received both treatment conditions, each in a different data collection session.

Participants attended two data collection sessions. Sessions were scheduled at least 3 days apart to ensure wash-out of tDCS effects. This time frame was selected based on previous literature as appropriate to wash out the effects of a single tDCS session (Erdoğan et al., 2023; Friehs et al., 2021; Hurley and Machado, 2018; Lukasik et al., 2018). Both sessions followed the same overall procedure, but the EEG calibration tasks were only conducted in the first data collection session.

Data collection sessions will be described in three stages: pre-training, training, and post-training.

#### 2.3.1 Pre-training stage

When the participants arrived at the Syracuse University Aphasia Lab, following the consent process, they were provided the FISS-A to complete with help as needed in order to collect demographic data regarding general fatigue. This help, provided by a licensed speech-language pathologist, included reading the items aloud and an explanation of the Likert scale structure. As the FISS-A examines general fatigue, the participants were instructed to think about their experiences during most days of the past week.

In order to later analyze task-based fatigue changes over the course of the session, the participants were then asked to rate their current level of fatigue on a scale from 0 to 100. This scale was defined for the participants as 0 referring to no fatigue and 100 referring to extreme fatigue.

During the first data collection session only, this was followed by collecting head measurements to ensure proper placement of the EEG head strap. Following placement of the EEG, the first data collection also included completion of the EEG calibration tasks as described above.

After ensuring proper impedance and calibration of the EEG, participants were instructed to place their head on the headrest in front of the computer on which the behavioral CPT task was displayed, and the room lights were turned off to maintain consistency between sessions and participants. Instructions for the behavioral task were presented both visually and auditorily by the researcher as many times as needed until the participant showed comprehension of the task.

Immediately prior to the onset of the first trial block of the CPT task, participants’ eye gaze was calibrated and validated to ensure proper tracking of their pupils. Following validation of the eye tracking software, the CPT task was started. Participants completed 6 blocks of 60 trials each, with a break provided between blocks. The participants determined the lengths of the breaks, but total testing time did not vary between participants or sessions.

#### 2.3.2 Training stage

Once the CPT task was completed, the EEG head strap was removed from the participant, and replaced by the tDCS head strap. tDCS administration, either active or sham, was started at the onset of the APT tasks, and continued for 20 min.

APT tasks were conducted in the same order for each session and participant, with three sustained attention tasks (1 sound, 2 sounds, 2-back matching) followed by two selective attention tasks (1 sound with distractors, and 2-back matching with distractors). The stimuli used in the attention tasks differed between sessions, with one session using animal sounds and pictures, and the other using everyday noises and abstract shapes. The use of two distinct categories of stimuli was done to minimize the learning effect on task performance, and category presentation order was counterbalanced.

APT tasks were completed independently, with a researcher monitoring auditorily from an adjacent room; participants were able to ask the researcher to re-enter the room in case of technical difficulties. The researcher entered the room 20 min into the training stage to turn off the tDCS device and then left again to allow the participants to complete the attention tasks. Metacognitive questions following each task were completed independently, and participants who had difficulty understanding the questions or how to respond to them were instructed to skip them to maximize time during tDCS administration spent on attention training tasks.

#### 2.3.3 Post-training stage

Following completion of the APT assigned program, the tDCS head strap was removed and replaced by the EEG head strap. EEG impedance was assessed, and once confirmed, the participants were instructed to place their head back in the head rest.

Room brightness conditions were matched to pre-training settings to mitigate any potential for the participants’ pupillary light reflex to confound pupillometry data. Following this, instructions for the CPT task were presented visually and auditorily again until participants were able to express comprehension of the task. Eye tracker calibration and validation was completed, and the participants engaged in the same sustained attention task as in the pre-training stage. Letter presentation order and ISI order within each block were randomized and different across all four CPT administrations.

Participants completed 6 blocks of 60 trials each. Breaks were provided between blocks, and break length was determined by the participants. Eye gaze calibration was confirmed following each break and before the onset of the first trial in each block.

Following the completion of the CPT program and the removal of the EEG head strap, participants were once again asked to rate their current level of fatigue on a scale of 0 to 100, with 0 referring to no fatigue, and 100 referring to extreme fatigue.

**Figure 1:**
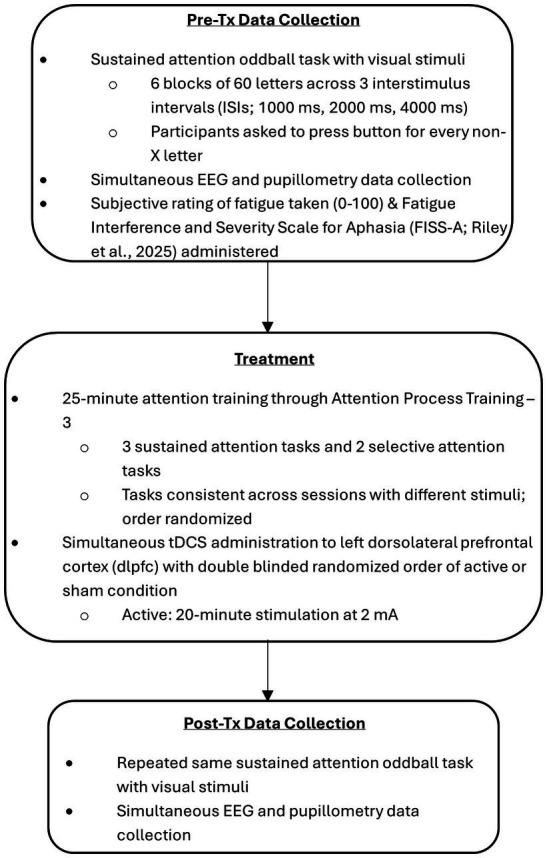
Study protocol.

Session procedure is illustrated in Figure 1.

### 2.4 Statistical analysis

Data were checked for normality across all outcome variables and was determined to violate the assumption of normality. As such, statistical analyses used in the current study were exclusively non-parametric.

Reaction time was collected for all trials, and analysis was conducted on the reaction times of correct non-X responses. This excluded empty responses due to non-reaction whether accurate (after the presentation of an “X”) or inaccurate as well as false positive responses to the presentation of an “X.” Included reaction times were averaged both by ISI and collapsed across ISIs for each of the 6 blocks as well as the entire task.

Reaction times as compared by ISI were analyzed via Friedman test comparing the four data collection periods for each ISI independently. This same statistical process was conducted for each of the 6 blocks, comparing the four data collection periods for each block independently. Reaction times for the entire CPT administration were compared solely by session; pre- and post-training administrations were compared to each other within a session using a Wilcoxon signed ranks test.

Pupil measurement was collected continuously over the course of four CPT administrations and averaged over the length of each trial such that 360 average pupil diameters were used in analysis. Pupil diameters were initially presented as arbitrary units and were converted to mm using a scaling factor based on an artificial pupil. Baseline data was calculated by ISI from the initial block of the first CPT administration, and all other pupil diameter trial averages subtracted this baseline to determine individual pupil dilation in mm.

Pupil dilation analysis was conducted for each ISI independent of each other. Initial Friedman tests compared each ISI across the four data collection periods. Post hoc analysis was conducted with Bonferroni adjustment for multiple comparisons (Chen et al., 2017) by dividing the alpha level of 0.05 by the number of tests, which in this case was 6. Thus, *p*-values determined from post hoc tests conducted via Wilcoxon signed ranks test were compared to an adjusted alpha level of less than 0.01.

The relationship between tDCS condition coded as a dummy variable and pupil dilation was modeled in a linear regression.

EEG data was reported for each trial over the CPT as the percentage likelihood the participant was in one of four attention states during that trial. The attention state used to classify each trial was the largest percentage likelihood reported. The number of trials classified as each attention state was analyzed as percentages of total trials both per individual blocks as well as over the entire CPT.

Friedman tests were used to analyze the percentage of trials spent in each attention state individually across all four data collection periods for each of the six blocks and the entire CPT.

Task-based fatigue was collected from participants rating their subjective rating of fatigue on a scale of zero to one hundred at the beginning and end of the sessions. Raw fatigue rating data was used in the analysis, with Wilcoxon signed ranks tests used to compare the pre- and post-training ratings for each session independently.

Effect size was calculated for the statistically significant results by dividing the absolute value of the *z* value by the square root of N (Pallant, 2020, p. 242).

## 3 Results

### 3.1 Effects on attention as measured through behavioral data

The behavioral measure of attention was operationalized for this study as reaction time of correct responses during the sustained attention CPT task. Within the oddball paradigm used for this study, correct responses refer to button presses following presentation of a non-X letter. False positives were not included in analysis of reaction time.

Nine participants were included in the analysis of these data, as 1 participant completed the CPT incorrectly, i.e., pressing the response button when X was presented and not pressing the response button for any other letter throughout all 4 task administrations.

#### 3.1.1 Average reaction time of entire CPT

Reaction time of correct responses was collected and averaged for all 360 trials for each of nine participants collapsed across ISIs. Reaction times were compared between pre-training and post-training CPT administrations for both conditions using a Wilcoxon signed ranks test. No significant differences were found for either condition. Reaction times and comparisons can be seen in Table 2.

**Table 2:** Reaction time in each block and the total CPT by condition.

	Pre-training (sham) reaction time (ms)	Post-training (sham) reaction time (ms)	Pre-sham & post-sham training *p*-value	Pre-training (active) reaction time (ms)	Post-training (active) reaction time (ms)	Pre-active & post-active training *p*-value	Friedman test
Block 1	467.37	451.22	0.26	458.02	444.98	0.52	0.39
*standard deviation:*	*72.92*	*85.12*		*128.42*	*75.34*		
Block 2	450.20	431.37	0.95	455.97	459.64	0.86	0.90
*standard deviation:*	*69.90*	*97.55*		*115.45*	*85.47*		
Block 3	457.88	452.33	0.59	443.30	457.12	0.52	0.77
*standard deviation:*	*87.42*	*72.25*		*107.76*	*76.16*		
Block 4	430.36	449.84	0.68	453.69	459.87	0.26	0.64
*standard deviation:*	*72.15*	*84.18*		*89.28*	*81.47*		
Block 5	459.04	460.18	0.77	463.63	486.00	0.37	0.74
*standard deviation:*	*72.15*	*82.34*		*97.016*	*75.74*		
Block 6	456.09	463.08	0.59	450.97	473.02	0.11	0.62
*standard deviation:*	*76.73*	*80.29*		*103.84*	*85.36*		
All blocks	453.50	451.47	0.17	454.36	463.35	0.59	0.39
*standard deviation:*	*67.68*	*77.82*		*104.80*	*77.01*		

Analysis between all four conditions conducted via Friedman test. Additional analysis of each session pre and post training conducted via Wilcoxon signed ranks test. No significant differences found. Standard deviations for means presented are denoted by italics.

Reaction times for the entire CPT were also compared by ISI; average reaction time for each ISI was compared across all four data collection periods via Friedman test. No significant differences were found for any of the ISIs: 1000 ms (*n* = 9, *p* = 0.71), 2000 ms (*n* = 9, *p* = 0.90) and 4,000 ms (n = 9, p = 0.71).

#### 3.1.2 Average reaction time across blocks of CPT

To look at the effect of time as a factor on reaction time and attention, average reaction times were also analyzed for each block collapsed across ISIs.

Freidman’s tests were conducted for each of the 6 blocks comparing the four testing conditions (pre or post training, active or sham tDCS). No significant differences were found for any of the 6 blocks, as seen in Table 2. Analyses of each session condition across time points were also conducted to investigate any non-significant trends in reaction time changes. No trends were determined.

Looking at the change in reaction time across the blocks for each condition independently and compared to the others does not reveal any notable patterns. This is consistent when looking at reaction time across blocks for individual participants; there were no observable patterns in reaction time changes as the blocks progressed.

### 3.2 Effects on attention as measured through pupil dilation changes

To look at physiological measures of attention, first pupil diameter changes during the sustained attention task were analyzed. Pupil data was reported by the eye tracker software in arbitrary units, which were converted using a scaling factor using an artificial pupil of constant size and measuring the distance maintained for all participants between their eye as held steady in the headrest, and the eye tracker camera.

Baseline pupil size was collected during the first block of the initial data collection session’s pre-training sustained attention task administration. Baseline pupil data were operationalized as the average pupil diameter in that initial block for each of the three ISIs. Pupil data used in the analysis consisted of the mean pupil diameter for each trial with the baseline pupil diameter for the respective ISI subtracted.

Pupil diameter changes for all ten participants were analyzed by ISI, comparing the average diameter change from baseline between the four testing conditions. Initial nonparametric analysis done via Friedman test revealed significant differences for all three ISIs, as shown in Table 3.

**Table 3:** Statistical analysis results for changes in pupil diameter relative to baseline for each ISI, compared by condition.

	1,000 ms	2000 ms	4,000 ms
Friedman test	0.01*	<0.01*	<0.01*
Pre-sham & post-sham training	0.29	0.14	0.20
Pre-sham & pre-active training	0.96	0.96	0.58
Pre-sham & post-active training	<0.01*	<0.01*	<0.01*
Post-sham & pre-active training	0.05	0.09	0.02
Post-sham & post-active training	0.02	0.02	0.01
Pre-active & post-active training	<0.01*	<0.01*	<0.01*

Significance denoted by *. Friedman test significance (α = 0.05). Post-hoc Wilcoxon signed ranks test with Bonferroni correction for multiple comparisons (α = 0.0083).

**Figure 2:**
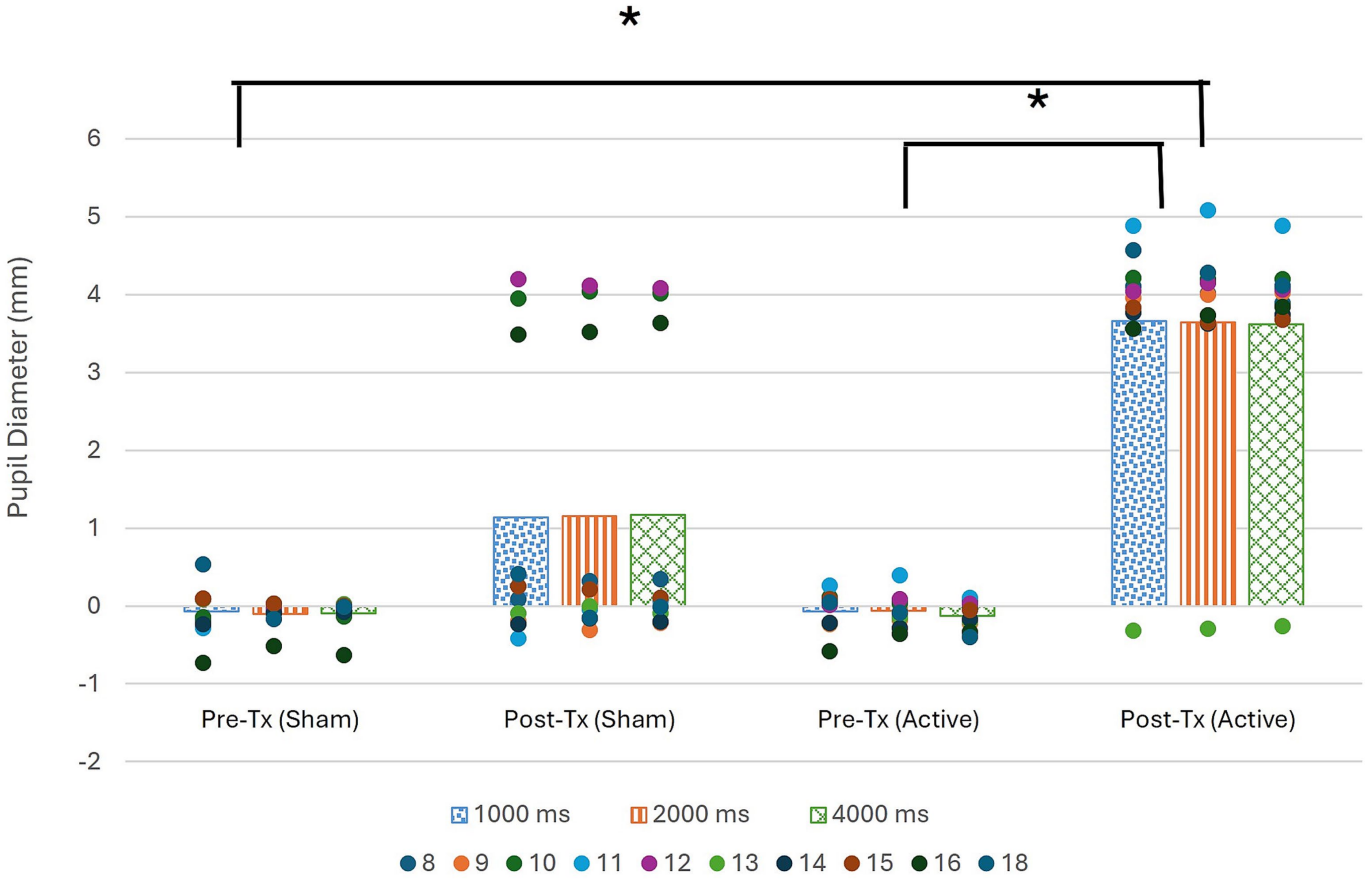
Pupil diameter during pre and post treatment data collection period as compared to baseline collected for each ISI at start of session. Significance denoted by * (*α* < 0.01).

*Post hoc* Bonferroni analysis corrected for multi-comparisons revealed significant differences found for all 3 ISIs between the pre-sham training testing period and the post-active training testing period, as well as the pre-active training testing period and the post-active training testing period, as seen in Table 3 and Figure 2. All statistically significant results (*p* < 0.01) have a large effect size (*r* = 0.85).

Figure 2 illustrates that in the post-active training data collection period, there is a significantly greater change in pupil diameter than in either of the pre-training data collection periods. The figure also reveals that the change in pupil diameter was positive, indicating pupil dilation occurred during the sustained attention task following active tDCS stimulation.

Association between tDCS condition and pupil dilation was assured via testing if tDCS condition significantly predicted pupil dilation operationalized as the average post-training pupil size collapsed across all three ISIs. The overall regression was statistically significant (R^2^ = 0.38, *F*(1, 18) = 10.91, *p* < 0.01). It was found that tDCS condition significantly predicted pupil dilation (*β* = 2.49, *p* < 0.01).

### 3.3 Effects on attention as measured through EEG

Physiological measurements of attention in this study also included EEG data collection. EEG data were reported in percent likelihood the participant was in one of four attentional states, no attention (NA), distracted attention (DA), moderate attention (MA), and high attention (HA), during the sustained attention task. EEG data were then analyzed by assigning the attentional state with the highest likelihood of occurrence to each trial.

**Figure 3:**
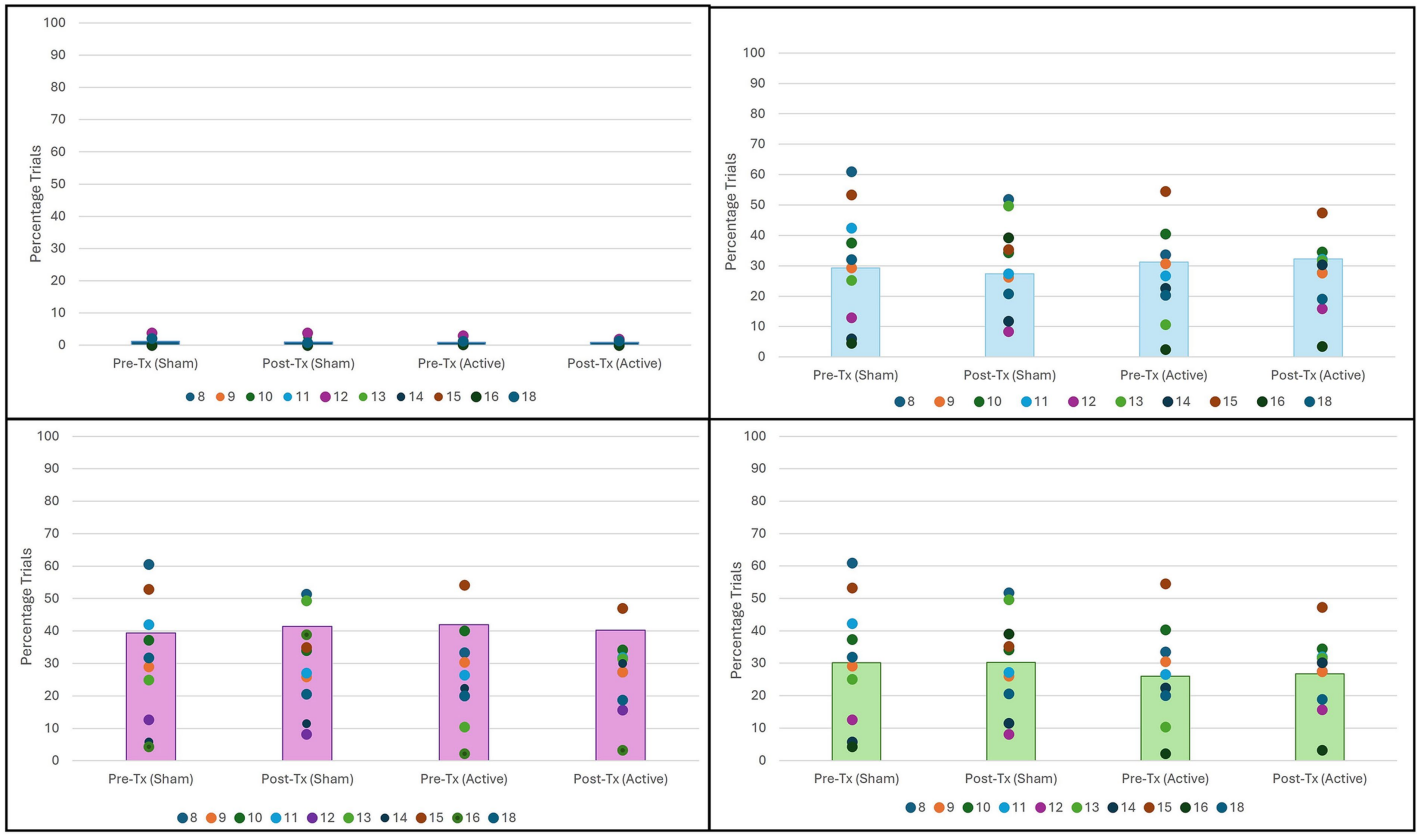
Percentage of trials in each attention state by condition. No significant difference found for any attention state. (Top Left) No attention by condition (*n* = 10, *p* = 0.83). (Top Right) Distracted attention by condition (*n* = 10, *p* = 0.20). (Bottom Left) Moderate attention by condition (*n* = 10, *p* = 0.94). (Bottom Right) High attention by condition (*n* = 10, *p* = 0.62).

The number of trials spent in each attentional state was compared via percentage of the complete sustained attention task, illustrated in Figure 3.

Comparison of attentional state percentages did not vary significantly between any state for any condition. However, as seen in Figure 3, in all four conditions, participants spent most trials in a state of moderate attention.

Time spent during the sustained attention task also did not affect the percentage of trials in each attentional state. Friedman test analysis of each block when compared across conditions revealed no significant differences as seen in Table 4.

**Table 4:** Percentage of trials in each block and the full task spent in each attention state by condition.

	Block 1	Block 2	Block 3	Block 4	Block 5	Block 6	All Blocks
No attention							
Pre-sham average:*standard deviation:*	1.02%*1.65*	1.14%*1.57*	1.02%*1.31*	1.27%*2.77*	0.95%*1.76*	1.46%*1.83*	1.17%*1.53*
Post-sham average:*standard deviation:*	0.70%*1.10*	1.02%*1.73*	1.27%*2.13*	1.09%*1.97*	1.46%*2.01*	0.83%*1.05*	1.04%1.29
Pre-active average:*standard deviation:*	1.02%*1.86*	1.14%*1.43*	1.02%*1.83*	0.63%*1.04*	0.69%0.91	0.51%*0.93*	0.93%*0.99*
Post-active average:*standard deviation:*	1.40%*1.81*	1.20%*1.17*	0.82%*1.20*	0.64%*0.79*	0.51%*0.50*	0.32%*0.81*	0.84%*0.86*
Friedman test	0.611	0.925	0.950	0.506	0.273	0.288	0.827
Distracted attention							
Pre-sham average:*standard deviation:*	30.63%*24.98*	30.85%*21.88*	29.83%*21.60*	28.36%*24.48*	32.01%*23.74*	32.80%*23.92*	29.30%*21.55*
Post-sham average:*standard deviation:*	31.55%*24.87*	29.41%*18.94*	30.08%*20.43*	25.52%*20.62*	29.88%*21.38*	25.61%*17.38*	27.32%*19.11*
Pre-active average:*standard deviation:*	31.46%*19.21*	31.99%*18.81*	30.06%14.78	34.55%*22.17*	36.14%*20.43*	33.87%*17.90*	31.21%*16.10*
post-active average:*standard deviation:*	30.32%*13.15*	33.30%*17.12*	33.53%*18.31*	37.17%*22.24*	33.81%*14.67*	34.46%*19.26*	32.35%*14.22*
Friedman test	0.879	0.668	0.518	0.321	0.468	0.841	0.197
Moderate attention							
Pre-sham average:*standard deviation:*	39.16%*23.44*	39.90%*15.41*	38.97%*15.39*	39.70%*14.37*	40.27%*17.71*	36.35%*12.52*	39.40%*13.78*
Post-sham average:*standard deviation:*	38.29%*17.45*	38.73%*13.53*	42.37%*15.58*	42.86%*9.57*	40.40%*12.62*	43.98%*9.25*	41.44%*9.89*
Pre-active average:*standard deviation:*	42.84%*20.30*	43.87%*18.52*	43.47%*17.21*	39.25%*17.16*	36.99%*15.44*	40.06%*14.04*	41.91%*14.47*
Post-active average:*standard deviation:*	41.24%*17.29*	40.91%*19.09*	38.99%*18.80*	37.12%*17.20*	39.93%*11.81*	39.29%*12.44*	40.20%*14.52*
Friedman test	0.989	0.564	0.530	0.743	0.689	0.210	0.948
High attention							
Pre-sham average:*standard deviation:*	29.13%*23.58*	28.11%*21.60*	30.19%*21.28*	30.66%*21.04*	26.77%*18.41*	29.38%*19.76*	30.13%*18.99*
Post-sham average:*standard deviation:*	29.46%*18.17*	30.84%*17.45*	26.28%*15.90*	30.54%*16.96*	28.26%*17.25*	29.58%*16.79*	30.21%*14.50*
Pre-active average:*standard deviation:*	24.67%*18.67*	23.00%*15.06*	25.45%*16.55*	25.57%*18.34*	26.18%*17.08*	25.57%*14.05*	25.95%*14.78*
Post-active average:*standard deviation:*	27.04%*17.12*	24.59%*14.80*	26.65%*16.26*	25.07%*15.24*	25.76%*11.68*	25.93%*13.55*	26.71%*11.88*
Friedman test	0.845	0.513	0.735	0.836	0.392	0.728	0.615

Friedman test conducted for each block and attention state. No significant difference was found. Standard deviation for each average percentage presented is denoted by italics.

### 3.4 Effects on subjective ratings of post-stroke fatigue

Fatigue was measured at the beginning and end of both sessions, with participants rating their current level of fatigue on a scale of 0 to 100. These ratings can be seen in Table 5. Only two participants indicated a decrease in fatigue from beginning to end, and these decreases only happened during the session wherein active stimulation was administered.

**Table 5:** Subjective ratings of fatigue collected at beginning and end of both sessions, separated by condition.

	Pre-sham stimulation	Post-sham stimulation	Pre-active stimulation	Post-active stimulation
P8	20	35	10	50
P9	0	60	0	0
P10	50	80	60	85
P11	40	67	0	35
P12	70	90	50	50
P13	0	0	**10**	**0**
P14	10	50	0	0
P15	10	20	**40**	**35**
P16	20	90	0	90
P18	0	0	0	0

Bold numbers indicate a decrease in fatigue.

Analysis of the effect of active tDCS administration as compared to sham on task-dependent fatigue was conducted via the Wilcoxon signed ranks test. Looking at the differences in fatigue rating for all participants, a significant difference was found between the pre- and post-sham stimulation ratings (*n* = 10, *p* = 0.01) with a large effect size (*r* = 0.80), while no significant difference was found between the pre- and post-active stimulation ratings (*n* = 10, *p* = 0.12).

**Figure 4:**
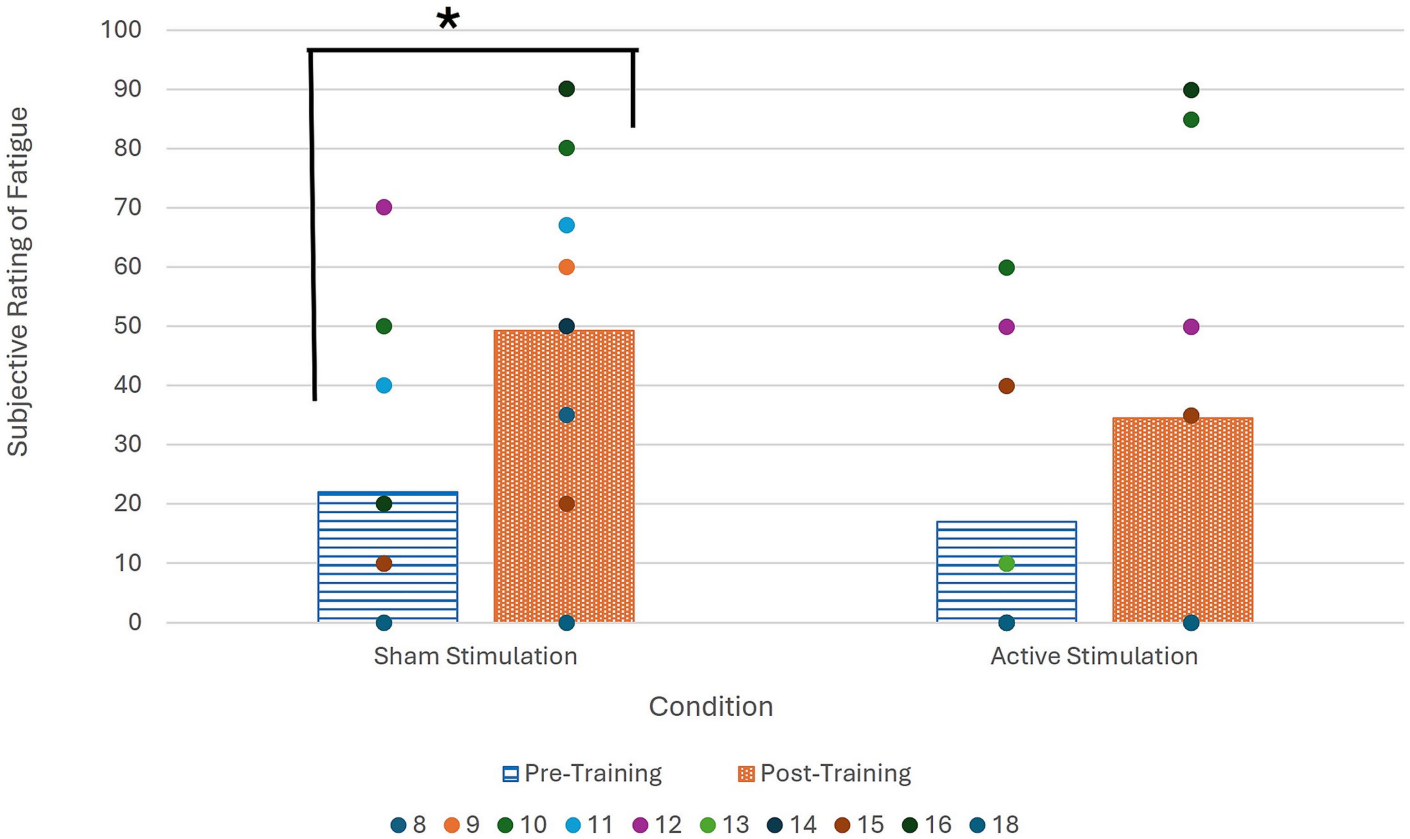
Average subjective fatigue ratings at beginning and end of both sessions for all participants. Wilcoxon signed ranks test significance denoted by * (α = 0.05; *p* = 0.01).

As seen in Figure 4, the post-sham fatigue ratings are higher than the pre-sham fatigue ratings, indicating a statistically significant increase in fatigue when no active stimulation was administered that was ameliorated by administration of active tDCS.

## 4 Discussion

The current study investigated the impact of a single session of tDCS on attention and fatigue, with a focus on physiological measurements of attention. This was achieved using EEG and pupillometry measures of attention as well as behavioral measures of attention and fatigue analyzed across four conditions: before and after receiving either active or sham transcranial direct current stimulation. The results showed an increase in pupil diameter following active stimulation that was not present following sham stimulation, but no significant change in attention as measured either through EEG or behaviorally through reaction time. Results also showed a significantly smaller session-related increase in fatigue for people who self-report milder levels of general post-stroke fatigue when participants received active stimulation.

The significant increase in pupil dilation during a sustained attention task following active tDCS reveals a measurable effect of this stimulation on the neurological underpinnings of attention, more specifically the LC-NE pathway (Bouret and Sara, 2004; Harley, 1991; McBurney-Lin et al., 2019). While this heightened activation secondary to active stimulation did not directly impact either EEG or behavioral responses during this task, it guides us to questions for further research on how to bridge that connection.

Locus coeruleus activation heightens norepinephrine production, which in turn increases levels of sustained attention and information processing capacity during tasks via neuromodulation of frontal–parietal control networks (Esterman and Rothlein, 2019). This suggests that by increasing LC activation, and thus increasing norepinephrine production, attention, and specifically task-based attention, will improve. This is also evidenced by previous studies denoting that disruptions in the LC-NE pathway are associated with attentional deficits (Zhang et al., 2023).

Also, the proxy evidence for LC activation following left DLPFC tDCS provides support to the connection between these brain regions, especially as it relates to attention and alertness (Mannarelli et al., 2015). There is also evidence to support the functional connectivity between the prefrontal cortex and the LC, and how this connection is associated with improved response inhibition (Tomassini et al., 2022), an executive functioning skill highly associated with higher accuracy in an oddball task (Zhao et al., 2023). This implies that increasing activation in the prefrontal cortex, such as through tDCS, will improve performance in a sustained attention task reliant on response inhibition, an implication strengthened by evidence associating stronger prefrontal neural connectivity with cognitive control and inhibition (Friedman and Robbins, 2022). The lack of measured improvement in the current study is thus notable and may be due to the limitation of stimulation being presented during only a single session. This reveals avenues for further research investigating the relationship between the length of stimulation and its effect on attention.

In spite of this, the stimulation presented to the left DLPFC leading to increased pupil dilation and the estimation of increased LC-NE pathway activation lends further support to the use of this stimulation as an adjuvant therapeutic tool for treating attention.

The lack of behavioral or EEG response change may lead to questions about the method of stimulation occurring during tDCS administration. Literature on tDCS has presented disparate theories on the path of activation, whether through directly activating cortical nerves, or indirectly activating brain regions through cranial nerves stimulated through the scalp or face (Majdi et al., 2024). In studies reporting that tDCS effects are mediated via peripheral nerves, evidence continues to show heightened activation of the LC following active stimulation. These studies also show behavioral effects of this activation, specifically on cognitive skills (Vanneste et al., 2020; Majdi et al., 2023) and as such it is likely that whether tDCS effects on the brain are direct or indirect, they should demonstrate similar results.

This may be counteracted by idiosyncrasies within the post-stroke population. Specifically, lesion size and location within this population may influence tDCS effects as mediated by peripheral nerve conductance. The tDCS montage used in the current study was consistent across participants, and lesion site was controlled for such that a lesion in the targeted region of the brain was an exclusionary factor. However, evidence has shown that lesioned tissue may directly impact electrical current flow (Evans et al., 2023; Krishnamurthy et al., 2025), which would cause individual differences in tDCS outcomes based on lesion characteristics. However, despite this, uniform tDCS montages have been shown to produce demonstrable effects in post-stroke individuals in various domains including motor skills, cognitive skills, and language (Gomez Palacio Schjetnan et al., 2013; Bornheim et al., 2020).

The locus coeruleus also plays a significant role in cognitive reserves and overall neurological health, especially while aging (Mather, 2020). LC activation has been associated with cognitive health and increases in cognitive reserve. Evidence has also been shown to suggest that LC activation and norepinephrine release due especially to mentally stimulating activities can be a protective factor for neuron health and density, thus aiding to prevent cognitive decline due to aging (Mather and Harley, 2016; Wilson et al., 2013). This is especially relevant in the post-stroke population, as stroke has been shown to increase cognitive decline in a way that is positively correlated with increasing age (Lo et al., 2022). This provides support for the use of tDCS, as a means for heightening LC activation, as an adjuvant therapy tool. Likely, sufficient therapy using tDCS would require multiple sessions; this method could be added to rehabilitative therapy such as speech and language therapy (Sebastian et al., 2016).

In determining clinical implications, regarding behavioral treatment paired with tDCS, research regarding effective LC activation has indicated that phasic LC firing is more effective in protecting against LC degradation (Omoluabi et al., 2021). Phasic LC firing, referring to high bursts of activation typically occurring over more steady tonic activation at the onset of novel, salient, or behaviorally relevant stimuli (Guedj et al., 2017), can be directly elicited through an oddball task (LoTemplio et al., 2021) such as the sustained attention task used in the current study.

Thus, with data from the current study suggesting that tDCS targeting the left DLPFC leads to heightened LC activation, even without direct improvements on attention via EEG or behavioral responses, there may be underlying improvement on overall cognitive health secondary to this treatment.

While the EEG data did not demonstrate a difference in attention state between conditions, the results provide additional support that PWA spend most of the time in a state of moderate attention while engaged in a non-linguistic task (Hibshman and Riley, 2024). There was no statistically significant difference between attentional states within any of the four conditions; however, there may be clinical significance found in the higher percentage of trials spent in the moderate attention state. Specifically, this supports evidence showing that non-linguistic attention tasks do not require more vigilant attention for PWA, which could be further elucidated in the context of clinical implications in future research (Hibshman and Riley, 2024).

Looking at individual patterns of attention state progression through the sustained attention task, some variance was noted, especially in the percentage of trials spent in either the high attention state or the distracted attention state. Individual differences in demographics or in effort and motivation maintenance during the duration of the task likely caused these periods of either heightened or lessened attention (Katz et al., 2016), and further research could be beneficial to investigate how these individual differences impact behavioral measures of attention.

Post-stroke fatigue is an area of growing research, especially in finding potential efficacious treatments (English et al., 2024). Within the limited number of participants included in this study, far reaching claims on the effect of tDCS on post-stroke fatigue cannot be made; however, individual differences in fatigue level can lead to tangible impacts on those individuals.

Two participants included in this study reported a decrease in task-based fatigue from the beginning of a session to the end, and both of these reported sessions occurred when active stimulation was provided. For these participants, tDCS appeared to counteract task-based fatigue.

Overall average task-based fatigue increased significantly when sham stimulation was provided, but that increase was not found when participants received active tDCS. These results support previous findings (Charvet et al., 2018; De Doncker et al., 2021) to suggest that active tDCS may be an effective treatment for task-based fatigue.

### 4.1 Limitations

One limitation in this study is the small number of participants, a limitation made especially stark due to the idiosyncratic nature inherent to aphasia as a disorder (Le et al., 2024). The effects of differences in aphasia severities and subtypes were minimized by the within-participant study design; however, the exclusion of one participant’s behavioral results due to difficulty understanding the task’s instructions demonstrates that there is a need for a wider range of severities to be included.

The number of participants also limited additional analysis of factors impacting these results such as lesion site and reported levels of general fatigue. Investigation of lesion site as a potential confounding demographic factor revealed a relative homogeneity in lesion location and stroke etiology, disallowing a more thorough investigation which might have been possible with more participants. Similarly, with only three participants reporting levels of general fatigue above the clinical marker on the FISS-A, *post hoc* analysis of the impacts of high levels of general fatigue were limited by insufficient data.

This limitation was also apparent in power analysis. *Post hoc* power analysis was conducted via g*power using effect size for primary analysis with statistical significance (i.e., pupil dilation following active stimulation) with power determined as 0.65. Sample size of 10 was used due to recruitment limitations, and post hoc power of this sample size is impacted by these limitations.

Another limitation is in the scope of this study; by focusing on the effects of a single session of tDCS, we may have missed physiological effects that may occur secondary to continued tDCS use. Also, when looking at fatigue and EEG recordings, analysis of tDCS effects could be better understood with more subjective information regarding each participant’s perceived effort and motivation.

### 4.2 Future research

The change in pupil diameter, and its implications for locus coeruleus activation, demonstrate that neurological underpinnings for attention may be positively impacted by tDCS, even after a single session. However, the lack of significant differences either in the EEG measures of attention, or the behavioral measures of attention, imply that the single session of tDCS is not sufficient to produce clinically significant changes in attention. Thus, a primary area of future research would be to look at the effects of multiple sessions of tDCS on physiological measures of attention.

This research could also be expanded to look at both non-linguistic tasks, such as the CPT used in the present study, as well as linguistic tasks. Expanding the scope of interest would allow for further understanding on how tDCS may impact attention across levels of attentional resource allocation.

This would also be a strong direction when looking at tDCS as a treatment for post-stroke fatigue, as investigating multiple sessions of tDCS may lead to stronger evidence for people experiencing mild fatigue, as well as providing relief of fatigue symptoms for those who self-report clinically significant fatigue as is demonstrated in previous studies (De Doncker et al., 2021).

Patient demographics is also an area of future research, specifically age and sex, which have been shown to impact responses to tDCS in previous studies (Bhattacharjee et al., 2022; Licata et al., 2023). With larger participant numbers, additional analyses can be conducted to investigate the role of age and sex on both physiological and behavioral effects of tDCS. Lesion location and reported levels of general fatigue would also be interesting factors to investigate in future studies, to further elucidate the potential individual differences in participants impacting responses to this treatment.

Another area of future research would be to use neuroimaging such as functional magnetic resonance imaging (fMRI) to investigate the direct impact of tDCS on the locus coeruleus and connected regions. Studies using both tDCS and fMRI have been able to see the effects of stimulation on the brain both during and after stimulation using concurrent and/or sequential study designs (Esmaeilpour et al., 2019).

Historically, because of the small size and depth of positioning in the brainstem, neuroimaging tools have had difficulty indexing the activity of the LC, leading to the use of pupillometry as a proxy measurement; although this has been made more applicable due to technological advances in the field, additional steps are still required to ensure validity (Poe et al., 2020; Laeng et al., 2012). Specifically, recommendations have included using the highest resolution possible and coregistering functional and structural images to ensure appropriate localization for individuals. Another suggestion is comparing fMRI data to indirect measures of LC activation such as pupillometry (Liu et al., 2017), making the addition of fMRI methodology to the present study a strong direction for future research, especially in consideration with the impacts of lesion characteristics on tDCS efficacy (Krishnamurthy et al., 2025).

## Data Availability

The raw data supporting the conclusions of this article will be made available by the authors, without undue reservation.
